# Molecular epidemiology of *Candida albicans* infections revealed dominant genotypes in waterfowls diagnosed with esophageal mycosis

**DOI:** 10.3389/fvets.2023.1215624

**Published:** 2023-06-29

**Authors:** Marianna Domán, László Makrai, Balázs Vásárhelyi, Gyula Balka, Krisztián Bányai

**Affiliations:** ^1^Veterinary Medical Research Institute, Budapest, Hungary; ^2^National Laboratory for Infectious Animal Diseases, Antimicrobial Resistance, Veterinary Public Health and Food Chain Safety, Budapest, Hungary; ^3^Department of Microbiology and Infectious Diseases, University of Veterinary Medicine, Budapest, Hungary; ^4^Veterinary Diagnostic Directorate, National Food Chain Safety Office, Budapest, Hungary; ^5^Department of Pathology, University of Veterinary Medicine, Budapest, Hungary; ^6^Department of Pharmacology and Toxicology, University of Veterinary Medicine, Budapest, Hungary

**Keywords:** *Candida albicans*, epidemiology, multilocus sequence typing, esophageal mycosis, population structure, waterfowls

## Abstract

Fungal infections of animals could yield significant economic losses, especially in the poultry industry, due to their adverse effects on growth, feed intake, digestion, and reproduction. Previous investigations showed that *Candida albicans* plays the main etiological role in the esophageal mycosis of birds. In this study, we used multilocus sequence typing (MLST) to determine the population structure and molecular epidemiology of *C. albicans* isolated from geese and ducks in Hungary. Interestingly, only three known genotypes were identified among investigated flocks, namely, diploid sequence type (DST) 840, DST 656, and DST 605, suggesting the intra-species transmission of these genotypes. Additionally, two novel allele combinations (new DSTs) were found that have not been previously submitted to the MLST database. Phylogenetic analysis of isolates revealed a close relationship between DST 656 and DST 605 as well as between the two newly identified genotypes (designated DST 3670 and DST 3671). Although isolates from birds belonged to minor clades in contrast with most human isolates, no species-specificity was observed. Poultry-derived isolates were group founders or closely related to group founders of clonal complexes, suggesting that *C. albicans* is exposed to lesser selective pressure in animal hosts. The increasing number of genetic information in the *C. albicans* MLST database could help to reveal the epidemiological characteristics and evolutionary pathways that are essential for disease prevention strategies.

## 1. Introduction

*Candida* spp. are common members of the normal microbiota of the digestive tract in humans and various animals and act as opportunistic pathogens. Predisposing factors such as tissue damage, immunosuppression, and prolonged or inappropriate antimicrobial therapy may result in the imbalance of the microbial community, promoting the overgrowth of *Candida* and other yeasts (1). Birds are particularly susceptible to gastrointestinal candidiasis, which has been observed in geese, ducks, broiler chickens, guinea fowls, pheasants, quails, pigeons, parrots, and birds of prey (2–9). The oral cavity and esophagus are the most frequently affected areas where mycotic lesions appear. Mycosis of the esophagus is characterized by the thickening of the mucosa with white to off-white, raised circular or rugose lesions that are peeled hardly from the mucosal surface. Clinical signs include poor growth, listlessness, indigestion, and roughness of feathers but these are not specific to candidiasis. The progress of candidiasis begins with *Candida* colonization of the esophageal mucosa typically limited to the stratum corneum or it may spread into the superficial stratum spinosum (1). *C. albicans* is the primary causative pathogen, however, other species (e.g., *C. parapsilosis*, *C. rugosa*) have also been associated with avian candidiasis (1, 10–12). *C. albicans* can transform between a variety of morphological types, such as yeast, pseudohyphae, and hyphae, and both mycelial and yeast forms may occur within the lesions. The transition from yeast to hyphal form indicates that the fungi entered the pathogenic state (1, 13).

Although the occurrence of avian candidiasis is sporadic, outbreaks have been reported causing significant economic losses for farms. In Hungary, a substantial proportion of exported poultry products are made from waterfowl with an outstanding significance of ‘foie gras’ (liver of a duck or goose fattened by force-feeding). In order to label the product as ‘foie gras’ the liver should weigh more than 300 g and 400 g for ducks and geese, respectively (14). Force-feeding with modern systems has increased the efficiency of liver production. Nevertheless, in case of inadvertent events, the direct mechanical damage caused by the feeding tube and extreme dilatation lead to the development of microlesions on the mucosal surface of the esophagus. As a result of infection-induced thickened mucosa, the esophagus becomes inflexible and may rupture during the force-feeding process, which may lead to the death of the animal. Through the lack of obvious clinical symptoms and the short force-feeding period (~2 weeks, afterward the birds are slaughtered), the diagnosis of esophageal mycosis is usually *postmortem*. Furthermore, antifungal drugs to treat mycoses in waterfowls have not been licensed in Hungary due to food safety issues, putting the focus of antifungal strategies on preventive measures. Despite severe economic losses, little is known about the role of fungal pathogens in the upper digestive tract in poultry, and information on the prevalence of mycosis and on the genetic diversity of strains is fairly limited. Multilocus sequence typing (MLST) is a widely used method in epidemiological investigations with the potential to analyze the genetic variation of microbial species (15–19). Moreover, associations of MLST genotypes with virulence, pathogenesis, and antifungal susceptibility have been also reported (20, 21). Therefore, determining the genetic characteristics of strains may facilitate the development of a more effective and economical disease prevention procedure.

## 2. Materials and methods

### 2.1. Sample collection and species-level identification

In 2020, esophagus samples from dead ducks and geese were collected from a poultry slaughterhouse in Békés county, Hungary. Experiments with or sampling from live animals were not performed. Otherwise, all methods were carried out in accordance with relevant guidelines and regulations. Animals originated from six different farms; in all, there were 194 geese and 330 ducks. Samples were selected for mycological analyses based on gross pathological findings, e.g., thickened mucosa with white circular or rugose lesions, curd-like pseudomembranous patches. Swab samples were collected representatively from the whitish areas of the esophageal mucosa of birds from each farm and cultured on Sabouraud dextrose agar (SDA; VWR International LLC, Hungary) supplemented with chloramphenicol and incubated at 37°C for 48 h. Colonization of the esophagus with yeasts was also investigated by culturing samples from healthy animals. Specimens from mechanically damaged areas in the esophageal mucosa of ducks were also taken and cultured. Primary cultures showing various colony morphologies were further inoculated on SDA until a pure culture was grown. White or cream-colored, high-convex yeast colonies were investigated by molecular method. Species identification was carried out by sequencing the internal transcribed spacer (ITS) region of fungal rDNA (2, 22). Briefly, genomic DNA was extracted after incubating isolates for 2 days using the Fungi/yeast genomic DNA extraction kit (Favorgen, Taiwan) in accordance with the manufacturer’s instructions. DNA samples were amplified by PCR with fungus-specific universal primers ITS1 (5′-TCCGTAGGTGAACCTGCGG-3′) and ITS4 (5′-TCCTCCGCTTATTGATATGC-3′) in a final volume of 15 μL, using a reaction mixture containing 1 μL fungal DNA, 2 μL 10x DreamTaq buffer, 0.5 μL dNTP (10 mM), 0.5 μL forward and reverse primers (10 μM each), 0.1 μL DreamTaq DNA polymerase (5 U/μL; Thermo Fisher Scientific, United States), and 10.4 μL distilled water. The PCR protocol used included denaturation at 95°C for 3 min, 40 cycles of 95°C for 30 s, annealing at 50°C for 30 s, extension at 72°C for 1 min, and a final extension step at 72°C for 10 min. Purified gene fragments were sequenced using ITS1 and ITS4 primers with BigDye Terminator v3.1. cycle sequencing kit (Thermo Fisher Scientific, United States) on an ABI Prism 3130 Genetic Analyzer (Applied Biosystems). Comparative sequence analyses were carried out by the BLAST sequence analysis tool.^1^ A histopathological examination of esophageal samples was carried out to confirm the fungal infection. Sections were stained with hematoxylin and eosin. PAS-reaction and Grocott-staining were used for more specific identification of mycelium in tissues. The histopathological slides were scanned with a Pannoramic Midi slide scanner instrument (3DHistech, Budapest, Hungary). The digital slides were analyzed, then representative pictures were taken highlighting the pathological process with the SlideViewer software (3DHistech, Budapest, Hungary) (2).

### 2.2. Allelic and cluster analysis of *Candida albicans* isolates

Seven housekeeping genes, namely, *AAT1a*, *ACC1*, *ADP1*, *MPIb*, *SYA1*, *VPS13*, and *ZWF1b*, were amplified and sequenced according to a previously published method for *C. albicans* MLST (15). Seven independent PCR amplifications were performed for each isolate. The primers used in PCR assays, their amplicon lengths, and experimental conditions were described in detail in Supplementary Table 1 (23). Further conditions used in PCRs and Sanger sequencing were the same as detailed above. After bi-directional sequencing of PCR products, modification and concatenation of sequences were performed as described by Odds and Jacobsen (24) since the base at each polymorphic site can be homozygous or heterozygous in diploid organisms. Heterozygosity was identified by the presence of two coincident peaks at the same polymorphic loci on both forward and reverse strands and the consensus sequences of seven loci of all isolates were defined (24). The allelic profile (allele number) for each gene and allele combination (diploid sequence type, DST) were identified and numbered by comparing the sequences with those available in the *C. albicans* MLST database.^2^ Sequences that did not match with any of the preexisting sequences in the database got novel allele and DST numbers from the curator.

The DNA sequences of the seven genes were concatenated into a single sequence and single nucleotide polymorphisms (SNPs) were converted as described by Tavanti et al. (25), enabling cluster analysis and clade definition of diploid sequence data. The genetic relationship between concatenated sequences was conducted by an unweighted pair group method with arithmetic averages (UPGMA) algorithm with a p-distance model supported by bootstrapping with 1,000 replications as implemented in MEGA v6 software (26). The demarcation of new clades was determined based on a previously published method: clusters with more than 10 DSTs were assigned to a clade (27). Clonal complexes (CCs), defined as groups of DSTs with six of the seven gene sequences being identical (single-locus variant analysis), were predicted by the goeBURST algorithm^3^ to investigate the population structure and evolutionary links between isolates (28). CCs were numbered starting from 0 (for the CC containing the most DSTs). DSTs belonging to a CC were all believed to be descended from the same founding genotype. DSTs that could not be assigned to any group were designated as singletons. The minimum spanning tree was also constructed from concatenated DST sequences using the GrapeTree software to illustrate the phylogenetic relatedness between our isolates and isolates deposited in the MLST database^4^ (29).

## 3. Results

No macroscopic lesions associated with mycosis were detected in the esophagus of birds originating from Farm 1 and Farm 3 (Supplementary Table 2). It was found that 12 out of 45 geese (27%) from Farm 2 and 23 out of 51 (45%) geese from Farm 4 showed moderate (Supplementary Figures 1A,B) or severe yellow-white lesions (Supplementary Figures 1C,D) on the esophageal mucosa. Histopathological examination of the infected esophagi of geese revealed yeast, pseudohyphae, and hyphae forms of fungal species (Supplementary Figure 2). It was also observed that 47 (25%) and 27 samples (19%) from ducks held in Farm 5 and Farm 6, respectively, exhibited some kind of lesions (Supplementary Table 2). However, unlike in geese, apparent pathological changes in the esophagi of ducks were uncommon and fungal cells were found only in a few sections. Instead, mechanical damage was more prevalent with embedded feeding stuff, which was also observed by histopathology (Supplementary Figures 3, 4) (2).

The most frequently isolated species from geese was *C. albicans* (*n* = 25). *Kazachstania bovina* (formerly *Candida bovina*) was also identified in two cases, while this yeast was the only fungal species that grew on SDA from one esophageal sample. Isolates AP7, AP8, AP25, MT3, MT46, and MT47 originated from healthy esophagi, indicating *Candida* colonization of birds, however, macro- or microscopic pathological changes did not develop. In ducks, *K. bovina* (*n* = 18), *Trichosporon* species (*n* = 6), and *Saccharomyces cerevisiae* (*n* = 2) were identified, whereas *C. albicans* (*n* = 4) was relatively rarely cultured from samples (Table 1). Species-level identification from sample GD/2 47 revealed that sequencing the ITS could not make a distinction between *T. insectorum* and *T. faecale* as the nucleotide identity of this region in the two species was 100% (2). Generated ITS sequences of *C. albicans* isolates were deposited in GenBank under the following accession numbers: OR058909-OR058937.

**Table 1:** Yeast isolates cultured from esophageal mucosa of waterfowls and their genetic characteristics.

Host	Isolate identifier	Location of farms	Fungal species	Genotype of isolates determined by MLST	UPGMA clade	Clonal complex by eBURST
Goose	AP 7	Békéscsaba	*Candida albicans*	DST 840	7	16
	AP 8		*Candida albicans*	DST 840	7	16
	AP 19		*Candida albicans*	DST 656	4	1
	AP 22		*Candida albicans*	DST 840	7	16
	AP 23		*Candida albicans*	DST 840	7	16
	AP 24		*Candida albicans*	DST 840	7	16
	AP 25		*Candida albicans*	DST 840	7	16
	AP 26		*Candida albicans*	DST 840	7	16
	AP 29		*Candida albicans*	DST 656	4	1
	AP 38		*Candida albicans*	DST 840	7	16
	AP 39		*Candida albicans*	DST 656	4	1
	AP 40		*Candida albicans*	DST 840	7	16
	AP 41		*Candida albicans*	DST 656	4	1
	AP 44		*Candida albicans*	DST 840	7	16
	AP 45		*Candida albicans*	DST 656	4	1
	OT 1/1	Gádoros	*Candida albicans*	DST 656	4	1
	OT 1/2		*Kazachstania bovina*	–	–	–
	OT 2		*Candida albicans*	DST 605	4	1
	OT 3		*Candida albicans*	DST 605	4	1
	OT 4		*Candida albicans*	DST 605	4	1
	OT 5/1		*Candida albicans*	DST 605	4	1
	OT 5/2		*Kazachstania bovina*	–	–	–
	OT 15		*Candida albicans*	DST 656	4	1
	OT 16/1		*Candida albicans*	DST 605	4	1
	OT 16/2		*Kazachstania bovina*	–	–	–
	MT 3	Csanádapáca	*Candida albicans*	DST 3670	14	14
	MT 46		*Candida albicans*	DST 840	7	
	MT 47		*Candida albicans*	DST 3671	14	14
	Tsa 29	Petőfiszállás	*Kazachstania bovina*	–	–	–	
	Tsa 30		*Kazachstania bovina*	–	–	–	
	Tsa 31		*Trichosporon japonicum*	–	–	–	
	Tsa 32		*Candida albicans*	DST 605	4	1	
	Tsa 33		*Kazachstania bovina*	–	–	–	
	Tsa 34		*Candida albicans*	DST 605	4	1	
	Tsa 35		*Kazachstania bovina*	–			
	Tsa 36		*Candida albicans*	DST 605	4	1	
	Tsa 37		*Kazachstania bovina*	–	–	–	
	GD/2 38		*Kazachstania bovina*	–	–	–	
	GD/2 39			*Trichosporon japonicum*	–	–	–	
	GD/2 40			*Kazachstania bovina*	–	–	–	
	GD/2 41			*Trichosporon japonicum*	–	–	–	
	GD/2 42			*Saccharomyces cerevisiae*	–	–	–	
	GD/2 43			*Saccharomyces cerevisiae*	–	–	–	
	GD/2 44			*Trichosporon japonicum*	–	–	–	
	GD/2 45			*Candida albicans*	DST 605	4	1	
	GD/2 46			*Kazachstania bovina*	–	–	–	
	GD/2 47			*Trichosporon insectorum/T. faecale*	–	–	–	Duck
	GD/2 48		Orosháza	*Kazachstania bovina*	–	–	–	
	GD/2 49			*Trichosporon japonicum*	–	–	–	
	GD/2 50			*Kazachstania bovina*	–	–	–	
	GD/1 51			*Kazachstania bovina*	–	–	–	
	GD/1 52			*Kazachstania bovina*	–	–	–	
	GD/1 53			*Kazachstania bovina*	–	–	–	
	GD/1 54			*Kazachstania bovina*	–	–	–	
	GD/1 55			*Kazachstania bovina*	–	–	–	
	GD/1 56			*Kazachstania bovina*	–	–	–	
	GD/1 57			*Kazachstania bovina*	–	–	–	
	GD/1 58			*Kazachstania bovina*	–	–	–

**Figure 1:**
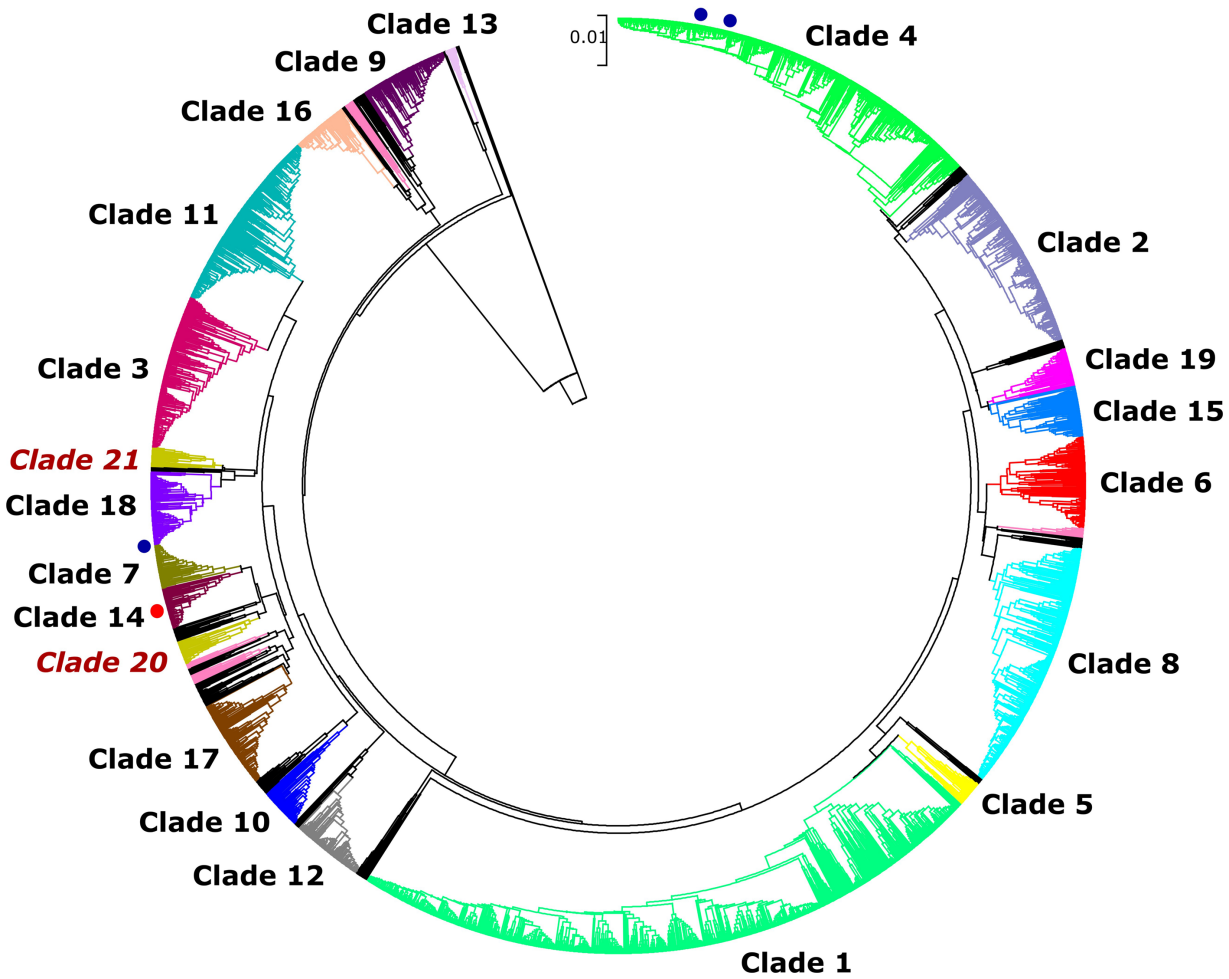
Unweighted pair group method with arithmetic averages (UPGMA) dendrogram generated from concatenated sequences of multilocus typing data of 3,690 *C. albicans* DSTs with p-distance method. DSTs obtained in this study are marked with blue (known genotypes) or red circles (novel genotypes).

**Figure 2:**
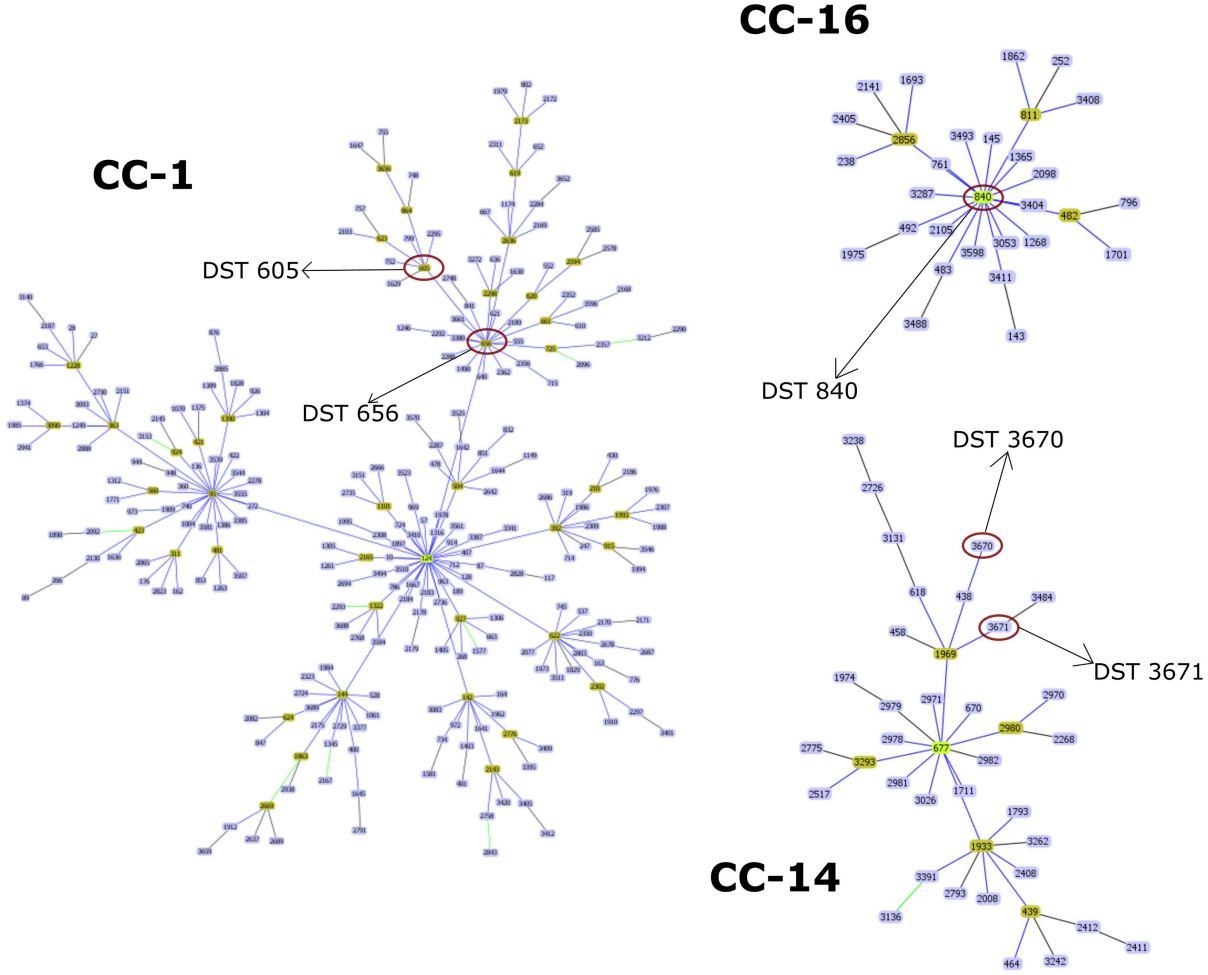
Population structure of *C. albicans* isolates constructed by goeBURST algorithm using diploid sequence types (DSTs) available in the MLST database. Clonal complexes (CCs) including DSTs of the current study (marked with a red circle) are shown. Putative group founder genotypes (light green) are positioned centrally in each cluster, while subgroup founders are shown in olive green.

**Figure 3:**
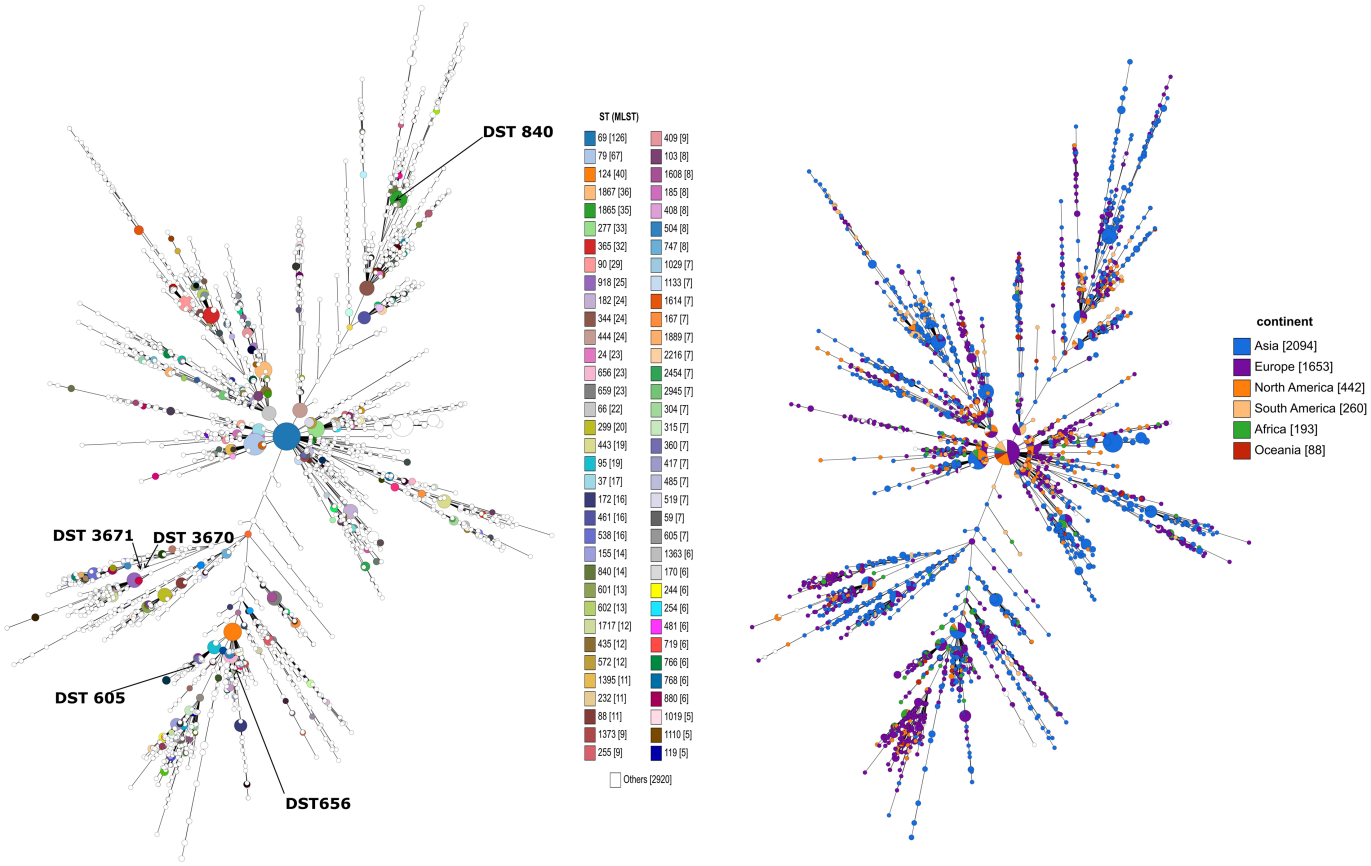
The minimum spanning tree illustrating the relationship between all *C. albicans* genotypes submitted to the public MLST database. Each circle corresponds to a specific DST. The size of the circle indicates the number of isolates belonging to a DST. In the picture on the right, the color of the circle represents the continent where the DST was isolated. The extended figure legend representing all DSTs can be found in Supplementary Figure S5.

Overall, 29 isolates of *C. albicans* were investigated in this study by the MLST method. Interestingly, only five genotypes were determined regardless of sample origin (Table 1). The dominant genotype was DST 840 (*n* = 11; 37.9%), followed by DST 605 (*n* = 9; 31%) and DST 656 (*n* = 7; 24.1%). All three genotypes were found in geese but only DST 605 was identified from ducks. Two of the examined isolates were assigned as new MLST genotypes (novel combination of previously described allele numbers) named DST 3670 (*n* = 1; 3.4%) and DST 3671 (*n* = 1; 3.4%), which was confirmed by the curator of the public MLST database. Both newly identified genotypes were derived from the mucosal sample of healthy geese. DST 840 (*n* = 10) and DST 656 (*n* = 5) were identified from geese in Farm 2, whereas *C. albicans* isolates from Farm 4 were genotyped as DST 605 (*n* = 5) and DST 656 (*n* = 2). *C. albicans* isolates originated from ducks in Farm 5 and Farm 6 belonged to DST 605 (*n* = 4). Cluster analysis of DSTs included in the present study along with all DSTs (*n* = 3,690) available in the database (25, 27, 30) revealed the phylogenetic relationship between animal and human isolates (Figure 1). The 29 *C. albicans* isolates were grouped into three clades. DST 840 clustered into Clade 7, DST 605 and DST 656 clustered into Clade 4, whereas new genotypes grouped together with isolates from Clade 14 (Figure 1). The most abundant clade was Clade 1, followed by Clade 4, Clade 8, Clade 2, Clade 3, and Clade 11. Clade 1–4 and Clade 11 were considered major clades with the most isolates reported globally, however, our analysis showed that Clade 8 has become a major clade as well. In addition, two new clades, named Clade 20 and Clade 21, were determined by UPGMA analysis. DSTs belonging to Clade 20 originated mainly from Europe (*n* = 23), but the clade also contained DSTs from America (*n* = 2), Asia (*n* = 9), and Oceania (*n* = 1). The DSTs in Clade 20 were isolated from different sources (animal, blood, feces, oral swab, oropharynx, sputum, vaginal swab), no correlation was observed between the clade and the site of isolation. Unfortunately, no data are available on DSTs belonging to Clade 21 except for DST 1835, which was isolated from China. However, most of the DSTs in this clade were denoted with consecutive numbers that may be serial isolates and raising the possibility of microevolution of *C. albicans* within patients. The evolutionary relationship of strains deposited in the MLST database was investigated based on allelic profiles by the goeBURST algorithm and the dataset was divided between 158 CCs and 1,148 singletons (Figure 2). DST 605 and DST 656 were grouped to CC-1 as putative subgroup founders. Of note, these two genotypes shared the same alleles except for *VPS13*, therefore DST 605 evolved presumably from DST 656. DST 840 was assigned to CC-16, which was predicted as the most probable ancestor of the cluster. The newly identified genotypes belonged to CC-14 and both of them developed from DST 1969 subgroup founder (Figure 2). Similarly to eBURST, the minimum spanning tree showed a diverse population structure of *C. albicans* isolates. DST 605 and DST 656 clustered together, whereas DST 840 was found distantly related to the other Hungarian DSTs. Moreover, DST 3670 and DST 3671 were genetically more closely related to each other than DST 605 and DST 656. Among previously known genotypes, DST 605 and DST 656 were isolated from Asia, Europe, and Africa. Additionally, an isolate genotyped as DST 656 was also reported from North America. DST 656 was detected from blood (South Africa, Mexico, United Kingdom, Kuwait, and South Korea) and chronic mucocutaneous candidiasis (United Kingdom). It was also reported from Israel, however, information regarding the origin of this genotype is not available. DST 605 originated from variable sources as it was identified from blood (United Kingdom, The Netherlands, Kuwait, and Israel), vaginal swabs (Morocco), sputum (Tunisia), and oropharynx (China). By contrast, DST 840 was detected only from samples from Asia and Europe. *C. albicans* genotyped as DST 840 was previously isolated from blood (Kuwait) and oral swabs/oropharynx (France and China). In addition, an isolate with unknown origin from Israel was also deposited in the MLST database (see text footnote 2). Overall, based on the analysis of sequences available in the *C. albicans* MLST database, minor genetic variations have evolved that are typical to some geographic areas. However, it cannot be ruled out that these DSTs are underrepresented due to the lack of data from some countries with available sequences (Figure 3).

## 4. Discussion

According to data collected by the Euro Foie Gras in 2018, the annual foie gras production in Hungary reached 1200–1400 tons and most of the first-class products (more than 90%) were exported to Western European and Far Eastern countries (30). Nevertheless, producing livers of the same size and the same quality is nearly impossible as several factors might affect production, such as age, sex, genetic factors, housing conditions, feeding preparation, the technology of force-feeding (mainly intensity), and personnel expertise (31). In addition, the development of esophageal mycosis leads to the deterioration of liver mass and liver quality, and even to the death of birds, yielding significant economic losses. In a previous study, we investigated the microbial causes of mycosis in the upper digestive tract in Hungarian waterfowls held for foie gras production in distinct flocks. The most prevalent opportunistic pathogen was *C. albicans* similar to that was found in other birds with crop mycosis (2).

The World Health Organization (WHO) developed the first fungal priority pathogen list in 2022, where *C. albicans* was categorized into the critical priority group along with *Cryptococcus neoformans*, *Candida auris,* and *Aspergillus fumigatus* due to their public health importance.^5^ Population-based investigations may promote overcoming knowledge gaps in colonization and transmission of *C. albicans* between variable hosts and reveal evolutionary relationships of genotypes. Data on fungal species colonizing the digestive tract of waterfowls are limited, therefore the identification of causative agents of esophageal mycosis is complicated. Although *C. albicans* has a wide host range and high environmental adaptability, studies mainly focus on human isolates and only a few studies are known that addressed the genotyping of avian *C. albicans* strains (4, 8). MLST has become one of the most reliable methods among established molecular typing techniques applied to determine the epidemiological and evolutionary relationship of pathogenic microorganisms due to its high reproducibility and sensitivity. We carried out a phylogenetic analysis of *C. albicans* using the MLST approach to investigate the epidemiological relationship and population structure of strains involved in etiologic causes of esophageal candidiasis. In all, 27 isolates were classified into three known genotypes (DST 840, DST 605, and DST 656) and we also identified two novel genotypes. Previously, we performed MLST data analysis of avian (goose, duck, falcon, and ostrich) and human *C. albicans* isolates originating from different sources. In that previous study, we determined several DSTs, however, we could not investigate genotype distribution between flocks. Among animal-derived *C. albicans* isolates, DST 840 has also been identified in association with esophageal mycosis (32). Overall, we explored the pattern of genetic variation in waterfowl-origin *C. albicans* populations and provided new information about the geographical distribution of genotypes by extending the data from Hungary. Currently, the *C. albicans* MLST database contains 63 isolates (including human and waterfowl-associated isolates) and 59 (93%) of these data were submitted by our research group, leading to some imbalance concerning the geographic origin of strains. This bias could be resolved by the submission of new data from other regions where the waterfowl industry is significant. Although isolates originating from animals are scant in the database compared to human-origin isolates (198 out of 5001; 3.96%), strains isolated from different sources were closely related to each other, and no host specificity was observed (33). The results of UPGMA and eBURST analysis correlated with each other and both revealed similar phylogenetic relationships between the examined strains. Odds et al. revealed that DST 69 is the most prevalent DST among *C. albicans* isolates submitted to the MLST database from all around the world, followed by DST 124 (27). With the expansion of the database, along with DST 79, these DSTs are still the most frequently determined genotypes globally (see text footnote 2). By UPGMA analysis, the majority of isolates in the database could be assigned to Clades 1, 2, 3, 4, and 11. Our dendrogram showed that the number of DSTs in Clade 8 has increased in recent years and Clade 8 is counted as another major clade. Additionally, two new clades were determined. Except for DST 605 and DST 656 (Clade 4, CC-1), our isolates belonged to minor clades (Clade 7, CC-16 and Clade 14, CC-14). Furthermore, the isolates obtained from poultry were group founders or closely related to group founders of CCs, suggesting that these genotypes were exposed to different selective pressures at a lesser extent than putative descendant genotypes. Another study of animal-derived isolates originating from camels further supported that isolates clustered in two clades and most DSTs were group- or subgroup founders (DST 69, DST 124, DST 142, DST 144) (34).

MLST could reveal associations between clades or CCs and antifungal resistance as it has been reported after analyzing *C. tropicalis* isolates collected from human blood samples (35). The UPGMA dendrogram and eBURST analysis of 48 *C. tropicalis* isolates causing candidemia in Thailand demonstrated that voriconazole- and fluconazole-resistant isolates and isolates with decreased susceptibility to posaconazole and itraconazole belonged to the same cluster. Although similar observations have not been reported yet with *C. albicans*, the constant extension of database and studies with non-human *C. albicans* isolates might contribute to a better understanding of the evolutionary routes of *C. albicans* (34).

In conclusion, since the majority of examined *C. albicans* isolates were assigned to three known DSTs, the transmission of this opportunistic pathogen between poultry might occur with contaminated feed or drinking water, resulting in minor outbreaks in certain flocks. UPGMA and eBURST analyses showed no host specificity of *C. albicans* DSTs and close genetic relatedness of poultry and human genotypes was observed, supporting the potential interspecies transmission. Of note, no associations between DSTs or MLST clades and the frequency of lesions have been reported yet in the case of esophageal mycosis in birds or humans. Lesions indicating esophageal mycosis of geese were frequently observed in some farms, whereas ducks seemed to be less susceptible to the disease. Due to representative sampling and the low sample size (*C. albicans* isolates), statistical analysis of our small dataset would give incoherent results. Therefore, further studies using a greater sample size are needed to statistically evaluate the molecular epidemiology of *C. albicans* infections in poultry. Experiences in veterinary practice showed that the treatment of esophageal mycosis is inadequate; thus various prevention measures should be implemented, including proper feeding and handling conditions and modernization of force-feeding technology, to reduce economic loss. Phylogenetic analyses using MLST data unveiled identical or closely related *C. albicans* genotypes as causative agents of esophageal mycosis of poultry in Hungary; however, further investigations are essential to determine the molecular background promoting the spread of these genotypes and to understand the links between colonizing and pathogenic state of yeasts.

## Data availability statement

The datasets presented in this study can be found in online repositories. The names of the repository/repositories and accession number(s) can be found in the article/Supplementary material.

## Ethics statement

Ethical review and approval was not required for the animal study because the experiments were carried out with dead animals as we sampled animals from a slaughterhouse. No live animals were involved in our study. Ethical review and approval was not required for the study on animals in accordance with the local legislation and institutional requirements.

## Author contributions

MD, LM, and KB: conceptualization. MD, LM, BV, and GB: investigation. MD: formal analysis and writing—original draft preparation. GB, LM, and KB: writing—review and editing. MD and GB: visualization. MD and KB: supervision and funding acquisition. All authors have read and agreed to the published version of the manuscript.

## Funding

This research was funded by the National Research, Development and Innovation Office of Hungary (NKFIH), grant number PD 128617, and the National Laboratory for Infectious Animal Diseases, Antimicrobial Resistance, Veterinary Public Health and Food Chain Safety, RRF-2.3.1-21-2022-00001.

## Conflict of interest

The authors declare that the research was conducted in the absence of any commercial or financial relationships that could be construed as a potential conflict of interest.

## Publisher’s note

All claims expressed in this article are solely those of the authors and do not necessarily represent those of their affiliated organizations, or those of the publisher, the editors and the reviewers. Any product that may be evaluated in this article, or claim that may be made by its manufacturer, is not guaranteed or endorsed by the publisher.

## Footnotes

^1^ https://blast.ncbi.nlm.nih.gov/Blast.cgi

^2^ https://pubmlst.org/organisms/candida-albicans

^3^ http://www.phyloviz.net/goeburst/

^4^ https://achtman-lab.github.io/GrapeTree/

^5^ https://www.who.int/publications/i/item/9789240060241
