# Supplementary material for: Molecular epidemiology of Candida albicans infections revealed dominant genotypes in waterfowls diagnosed with esophageal mycosis
Source: Front Vet Sci. 2023 Jun 29;10:1215624. doi: 10.3389/fvets.2023.1215624 (PMC10344593; doi:10.3389/fvets.2023.1215624)
Supplement: Supplementary file 1 [file Data_Sheet_1.docx]

Supplementary Material

Molecular epidemiology of *Candida albicans* infections revealed dominant genotypes in waterfowls diagnosed with oesophageal mycosis

Marianna Domán*, László Makrai, Balázs Vásárhelyi, Gyula Balka and Krisztián Bányai

*** Correspondence:** Marianna Domán: doman.marianna@vmri.hu

**Supplementary table 1.** List of gene fragments and primers used for *C. albicans* MLST analysis

| **Gene** | **Gene product** | **Primer sequence** | **Amplicon size (bp)** | **Annealing temperature (°C)** |
| --- | --- | --- | --- | --- |
| *AAT1*a | Aspartate aminotransferase | Fwd 5'-ACTCAAGCTAGATTTTTGGC-3'  Rev 5'-CAGCAACATGATTAGCCC-3' | 478 | 45 |
| *ACC1* | Acetyl-coenzyme A carboxylase | Fwd 5'-GCAAGAGAAATTTTAATTCAATG-3' Rev 5'-TTCATCAACATCATCCAAGTG-3' | 519 | 43 |
| *ADP1* | ATP-dependent permease | Fwd 5'-GAGCCAAGTATGAATGATTTG-3'  Rev 5'-TTGATCAACAAACCCGATAAT-3' | 537 | 45 |
| *MPIb* | Mannose phosphate isomerase | Fwd 5'-ACCAGAAATGGCCATTGC-3'  Rev 5'-GCAGCCATGCATTCAATTAT-3' | 486 | 46 |
| *SYA1* | Alanyl-RNA synthetase | Fwd 5'-AGAAGAATTGTTGCTGTTACTG-3'  Rev 5'-GTTACCTTTACCACCAGCTTT-3' | 543 | 46 |
| *VPS13* | Vacuolar protein sorting protein | Fwd 5'-TCGTTGAGAGATATTCGACTT-3'  Rev 5'-ACGGATGGATCTCCAGTCC-3' | 741 | 46 |
| *ZWF1*b | Glucose-6-phosphate dehydrogenase | Fwd 5'-GTTTCATTTGATCCTGAAGC-3'  Rev 5'-GCCATTGATAAGTACCTGGAT-3' | 702 | 45 |

**Supplementary table 2*.*** Occurrence of oesophageal lesions originated from geese and ducks held in different flocks

| **Origin of oesophageal samples** | **Number of samples** | **Cases of mucosal lesions (frequency)** |
| --- | --- | --- |
| Farm 1 | 49 geese | - |
| Farm 2 | 45 geese | 12 (27%) |
| Farm 3 | 49 geese | - |
| Farm 4 | 51 geese | 23 (45%) |
| Farm 5 | 185 ducks | 47 (25%) |
| Farm 6 | 145 ducks | 27 (19%) |


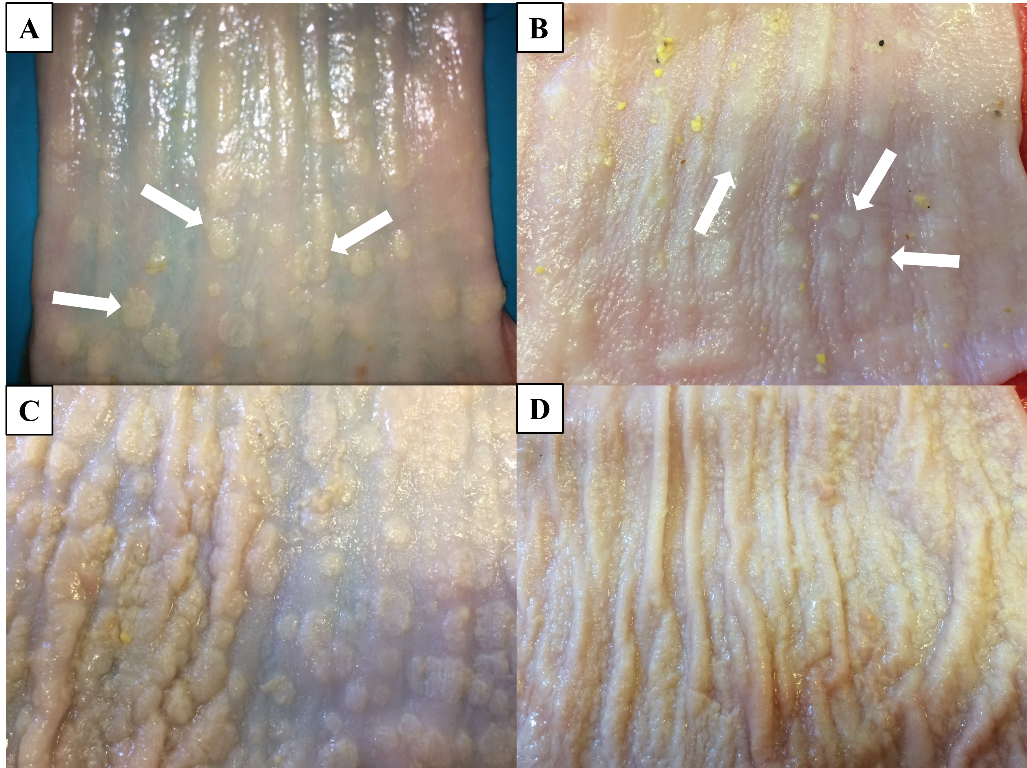


**Supplementary figure 1.** Different stages of mycosis characterised by whitish, thickened areas on the oesophageal mucosa of geese caused by yeasts. A-B: moderate oesophageal mycosis, C-D: severe oesophageal mycosis. White arrows indicate the yellowish-white lesions with varying size on the mucosal surface.


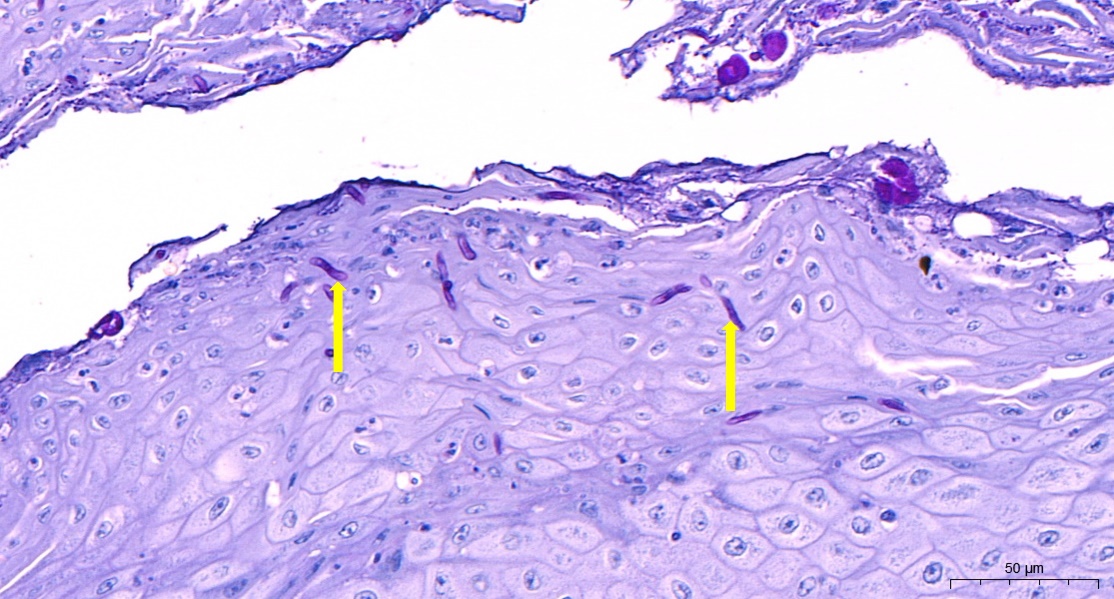


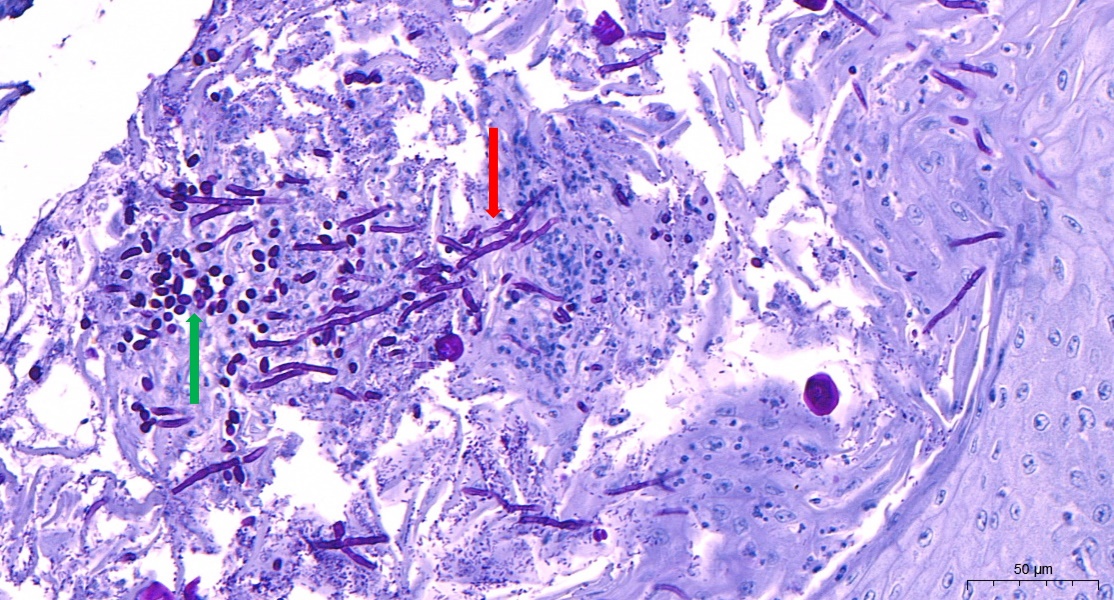


**Supplementary figure 2.** Histopathologic slide prepared from goose oesophagus. In the upper picture the yellow arrow indicates a pseudohyphae in the hyperplastic esophageal epithelium, whereas in the lower picture fungal hyphae are indicated with red arrow and yeast cells with a green arrow. Periodic acid–Schiff *(*PAS*)* staining, 90×, bar=50 µm 102×, bar=50 µm.


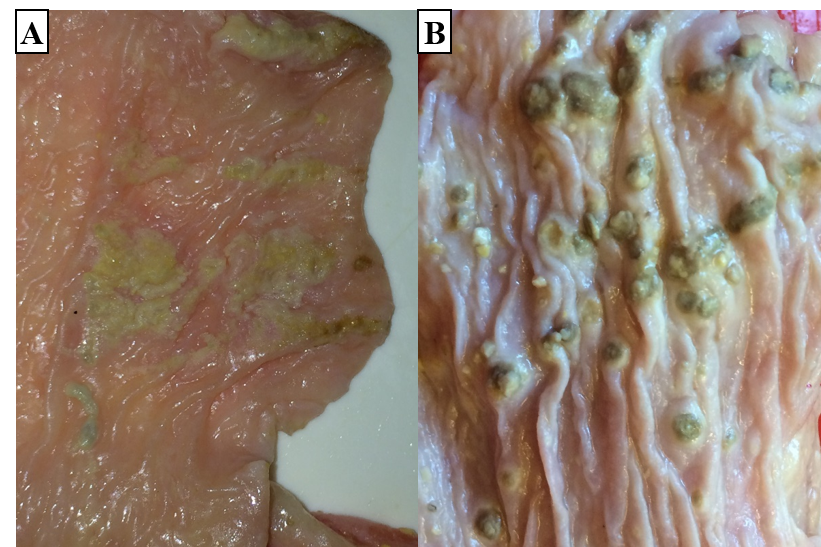

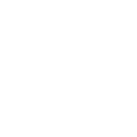

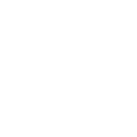

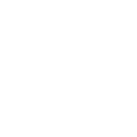


**Supplementary figure 3.** Moderate lesions (asterisks) (A) and mechanical damage with embedded feeding stuff (arrows) (B) on the mucosal surface of duck oesophagus (with the permission of the publisher) [2].

**
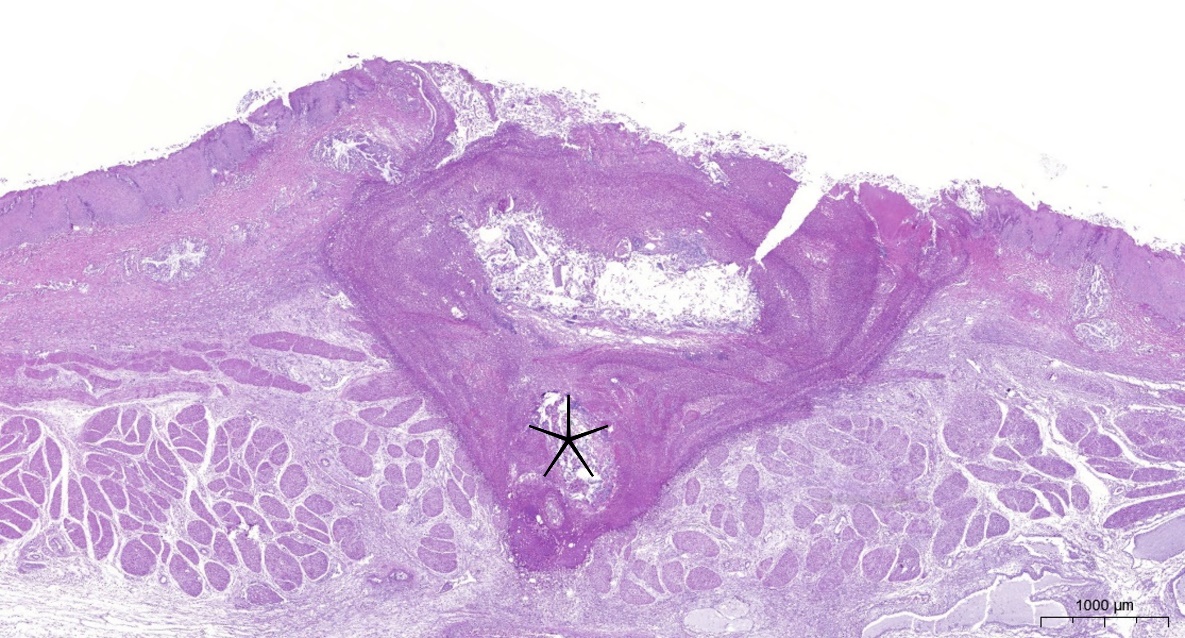
**

**
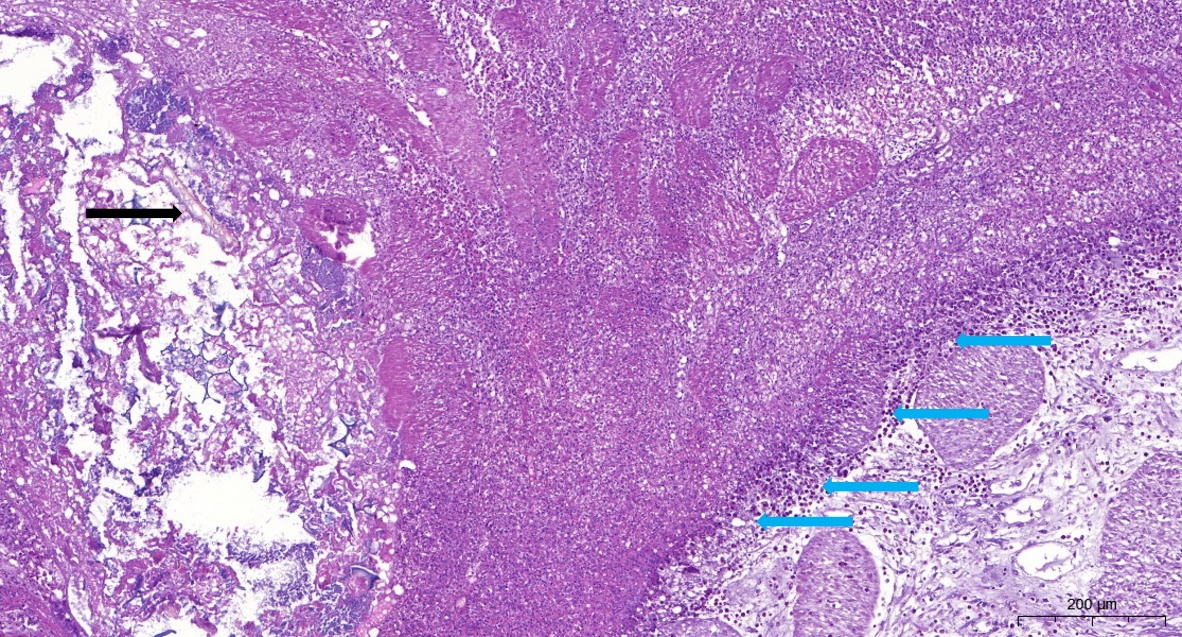
**

**Supplementary figure 4.** Histopathologic slide prepared from duck oesophagus. In the small power view picture deep mechanical oesophageal injury can be seen extending into the muscular layer (asterisk) in which feed particles of plant fibres are impacted (black arrow), and it is surrounded by a massive layer of inflammatory cells (blue arrows). Hematoxylin-eosin staining, 4×, bar=1 mm 24×, bar=200µm.


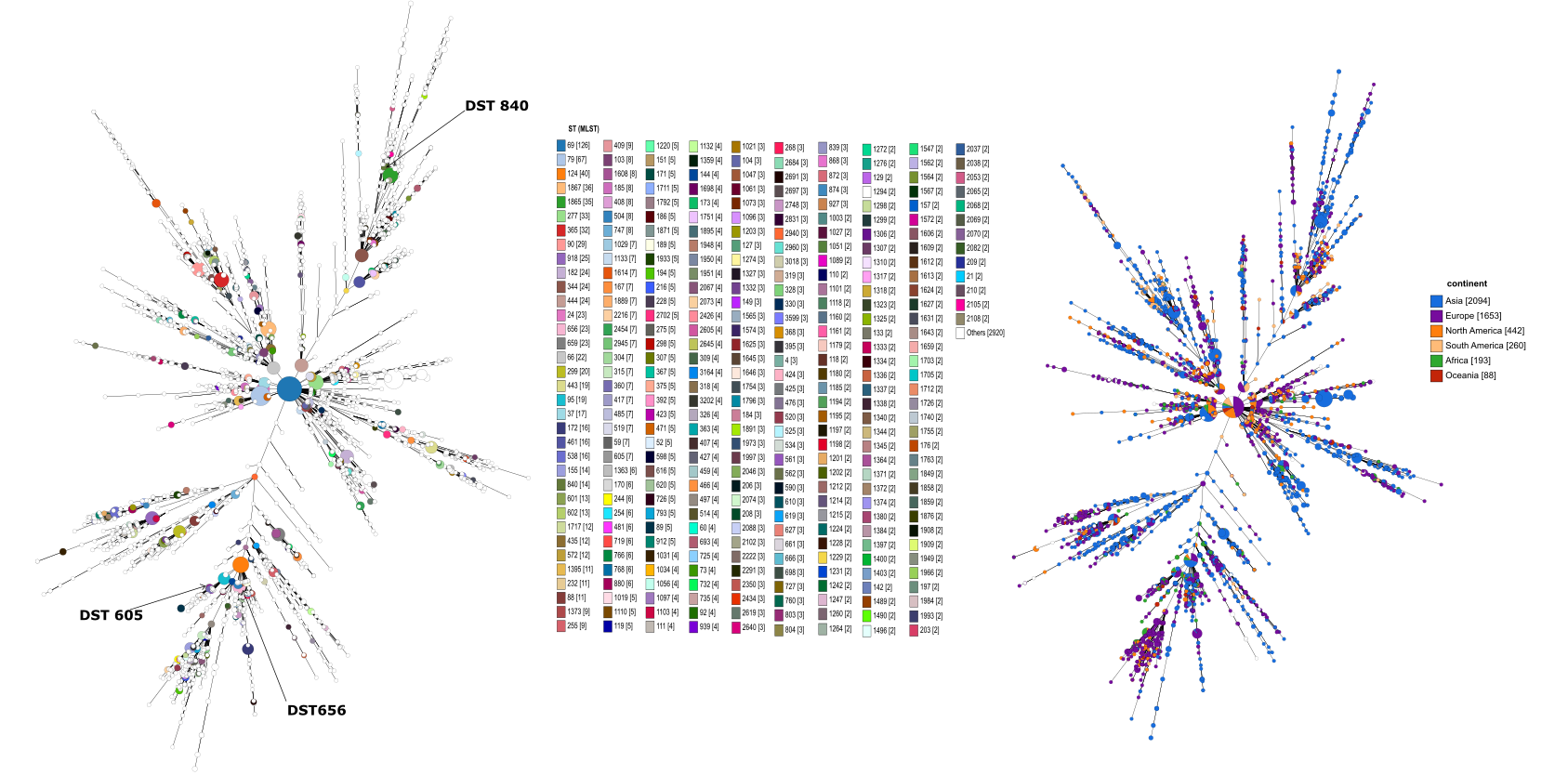


**Supplementary figure 5.** The minimum spanning tree illustrating the relationship between all *C. albicans* genotypes submitted to the public MLST database (with extended figure legend). Each circle corresponds to a specific DST. The size of the circle indicates the number of isolates belonging to a DST. In the right picture, the color of the circle represents the continent where the DST was isolated.
